# Identification of novel compound heterozygous variants in the *PEX10* gene in a Han-Chinese family with *PEX10*-related peroxisome biogenesis disorders

**DOI:** 10.1371/journal.pone.0322137

**Published:** 2025-04-23

**Authors:** Xiangjun Huang, Xinyue Deng, Xiong Deng, Hongbo Xu, Hao Deng, Lamei Yuan

**Affiliations:** 1 Department of General Surgery, the First Affiliated Hospital of Hunan University of Chinese Medicine, Changsha, China; 2 Xiangya School of Medicine, Central South University, Changsha, China; 3 Center for Experimental Medicine, the Third Xiangya Hospital, Central South University, Changsha, China; The Islamia University of Bahawalpur, PAKISTAN

## Abstract

The peroxisome biogenesis disorders (PBDs) are a group of rare inherited autosomal recessive diseases characterized by motor and cognitive neurological dysfunction, hypotonia, seizures, feeding difficulties, retinopathy, sensorineural hearing loss, hepatic and renal abnormalities, and chondrodysplasia punctata of long bones, and the clinical expression is variable. Exome sequencing and Sanger sequencing were used to identify the genetic defect for PBDs in a two-generation non-consanguineous Han-Chinese pedigree. Compound heterozygous variants, a novel splicing variant c.113-2A>G and a reported substitution c.890T>C (p.Leu297Pro), in the peroxisomal biogenesis factor 10 gene (*PEX10*) were detected. The splicing variant c.113-2A>G led to a canonical splice acceptor site inactivation, exon 2 skipping, and in-frame deletions (p.Ala39_Gly65del). The three patients had similar phenotypes of milder PBDs, which were further genetically determined as PBD6B. The findings extend the *PEX10* variant spectrum and may provide new insights into PBDs causation and diagnosis, with implications for genetic counseling and clinical management.

## Introduction

The peroxisome biogenesis disorders (PBDs) are a group of rare inherited autosomal recessive diseases characterized by neurological and developmental dysfunction with multisystem involvement, manifesting as hypotonia, seizures, mental retardation, feeding difficulties, visual and hearing impairment, and abnormalities in face, skeleton, liver, and kidney, with variable clinical expression [1–6]. According to the clinical manifestations, PBDs can be divided into two main groups, Zellweger spectrum disorders (ZSDs) and rhizomelic chondrodysplasia punctata type 1 (RCDP1). ZSDs include the mild infantile Refsum disease (IRD), the less severe neonatal adrenoleukodystrophy (NALD), and the most severe Zellweger syndrome [1,7–9], in which all three are accompanied by variable neurodevelopmental delay, hearing and vision impairment, and liver dysfunction in the first few months after birth [10]. Zellweger syndrome patients generally die within the first year of life, and NALD and IRD patients may survive to childhood and adulthood [11,12]. Approximately 80% of PBDs patients show phenotypes of ZSDs, and 20% cases have RCDP1 [12,13]. The estimated birth incidence is 0.002% for ZSDs and 0.001% for RCDP1 worldwide, with varied prevalence for both conditions among different populations [14]. Pathological defects of PBDs include impaired peroxisome assembly and function, relating to decreased peroxisome numbers, morphologically abnormal peroxisomes, and impaired peroxisomal alpha- and beta-oxidation, plasmalogen synthesis, and catalase distribution, though the exact mechanism remains unclear [8,14–16].

PBDs are primarily caused by biallelic variants in any of 14 different peroxisomal biogenesis factor (*PEX*) genes, *PEX1*, *PEX2*, *PEX3*, *PEX5*, *PEX6*, *PEX7*, *PEX10*, *PEX11B*, *PEX12*, *PEX13*, *PEX14*, *PEX16*, *PEX19*, and *PEX26* [1,14]. The *PEX10* gene pathogenic variants may be the fifth most common cause of PBDs (3.4%), following *PEX1* (48.5%), *PEX7* (17.7%), *PEX6* (13.1%), and *PEX12* (5.9%) based on the reported data worldwide. Laboratory findings of impaired peroxisomal functions and identification of pathogenic variants in genes like *PEX* are valuable and helpful for definite disease diagnosis [11].

This study sought to reveal the genetic factor giving rise to PBDs in a two-generation non-consanguineous Han-Chinese family. Novel compound heterozygous variants, c.113-2A>G (p.Ala39_Gly65del) and c.890T>C (p.Leu297Pro), in the *PEX10* gene (NG_008342.1, NM_153818.2), were identified, which may disrupt peroxisome biogenesis and therefore decrease metabolism, leading to multisystem abnormalities [17].

## Materials and methods

### Participators and clinical examination

**Fig 1:**
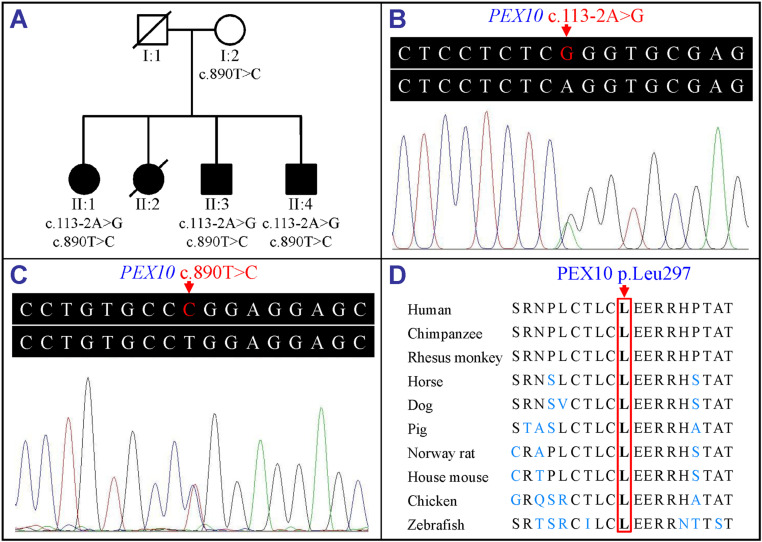
The c.113-2A>G and c.890T>C variant in the *PEX10* gene in a family with PBDs. (A) Family pedigree of the patients with autosomal recessive PBDs. White square with slash symbol indicates deceased unaffected male family member. White circle symbol represents unaffected female family member. Black circles and black squares denote affected females and males, respectively. Black circle with slash symbol indicates deceased affected female family member. The *PEX10* gene variants were indicated under the individuals who had genetic analysis. (B) The *PEX10* gene sequence with heterozygous c.113-2A>G variant of patient II:1. (C) The *PEX10* gene sequence with heterozygous c.890T>C variant of patient II:1. (D) Conservative analysis of the PEX10 leucine residue at position 297 (p.Leu297). *PEX10*, the peroxisomal biogenesis factor 10 gene; PBDs, peroxisome biogenesis disorders.

A two-generation Han-Chinese family with PBDs was recruited at the First Affiliated Hospital of Hunan University of Chinese Medicine, Changsha, China, from June to August, 2023 (Fig 1). Detailed clinical medical histories were collected, and physical, biochemical, and radiological examinations were performed on all participants. Available peripheral venous blood was sampled. Skin biopsy for fibroblast culture and peroxisomal functional analysis was not performed due to the refusal of the family. Written informed consent was obtained from each participant or the legal guardian. This study had received approval from the Institutional Review Board of the Third Xiangya Hospital, Central South University, Changsha, China, which was implemented from June 2023 to February 2025.

### Exome capture and sequencing

Genomic DNA (gDNA) was isolated from sampled peripheral blood lymphocytes via a phenol/chloroform extracting protocol. Exome sequencing was performed to unveil shared genetic causation of PBDs in two affected siblings (II:1 and II:3) of the pedigree by the established method of BGI-Shenzhen (Shenzhen, China) [18]. The “A” base and adaptor were ligated to the fragmented DNA sequentially, and ligated-PCR was used for amplification. Hybridization to the exome array for enrichment was conducted, followed by circularization and amplification for library. The SureSelect^XT^ Human All Exon V6 (Agilent Technologies, Inc., Santa Clara, CA, USA) was used for exome capture, and BGISEQ-500 platform was used to sequence the qualified circular library.

### Read mapping and variant analysis

The human reference genome (hg19) was used, and sequencing data were aligned using Burrows-Wheeler Aligner (v0.7.15). Genome Analysis Toolkit (v3.7), Picard (v2.5.0), and SnpEff tool were used for variants’ calling and annotation, including single nucleotide polymorphisms (SNPs) and insertions-deletions (indels) [19]. Common variants or non-pathogenic variants were filtered out using the human gene variant databases, including the Single Nucleotide Polymorphism database (dbSNP, version 141) and 1000 Genomes Project. Variants were further analyzed using the in-house exome databases with 3275 Chinese controls, as well as the National Heart, Lung, and Blood Institute (NHLBI) Exome Sequencing Project (ESP) 6500, Genome Aggregation Database (gnomAD), China Metabolic Analytics Project (ChinaMAP), Human Gene Mutation Database (HGMD), and the ClinVar database. The shared potential disease-causing variants were prioritized. PCR amplification was conducted with the gDNA samples and the designed locus-specific primers, and the presence of potential causative variants was further tested by Sanger sequencing on an Applied Biosystems 3730xl genetic analyzer (Applied Biosystems, Thermo Fisher Scientific Inc., Waltham, MA, USA) [20,21]. The primer sequences were as follows: 5′-CGGAGACAGAAGCAGAGAGG-3′ and 5′-TACCTGCAAGTGTGGTGAGG-3′ for variant 1, 5′-TAAGGTGCACCCACCTTGAC-3′ and 5′-CCACCTCACCTTGCTGCT-3′ for variant 2. Bioinformatics tools including Polymorphism Phenotyping version 2 (PolyPhen-2), Functional Analysis through Hidden Markov Models (FATHMM, v2.3), MutationAssessor, MutationTaster2021, Combined Annotation Dependent Depletion (CADD, v1.6), Berkeley Drosophila Genome Project (BDGP) Splice Site Prediction by Neural Network (v0.9), NetGene2 (v2.4), SpliceAI, and Pangolin were used to evaluate whether amino acid substitutions affected protein structures and functions and the variants’ effects on splicing [22–25].

### RNA extraction and analysis

To further determine the variants’ effects, total RNA was extracted from the peripheral venous blood of the unaffected mother (I:2) and three patients (II:1, II:3, and II:4) using the RNA-Solv reagent (Omega Bio-Tek Inc., Norcross, GA, USA). Reverse transcription for high-efficient complementary DNA (cDNA) synthesis was performed using the ReverTra Ace qPCR RT Master Mix with gDNA Remover (Toyobo Co., Ltd., Osaka, Japan), and further PCR amplification was completed using the following primer pairs for confirming the variants’ effects: 5′-GGCGCAGAAGGACGAGTA-3′ and 5′-CTGCCTGAAACCGTACAGC-3′ for variant 1, 5′-GAAGGAGTGGAGGCTGCA-3′ and 5′-GAGCTTCTGGGGAGGGAAC-3′ for variant 2. PCR fragments were analyzed by electrophoresis, separation, retraction (only for further evaluation of the splicing variant), and purification, and then sequenced on the genetic analyzer [26]. By comparing the areas under the peaks (AUP) of wild-type and mutant alleles, the nonsense-mediated mRNA decay caused by the variant was evaluated using the direct sequencing results of PCR fragments. The ImageJ software v1.54d (National Institutes of Health, USA) was applied to quantify the AUP, and Microsoft Excel 2021 (Microsoft Corp., Redmond, WA, USA) and GraphPad Prism v8.2.1 (GraphPad Software, LLC, Boston, MA, USA) were used for statistical analysis, in which Student’s *t* test was applied and *P*<0.05 was regarded as statistically significant [27]. For further checking the correct splicing caused by the potential splicing variant, primers for amplifying fragments covering both variants were designed, 5′-GCGCAGAAGGACGAGTACTA-3′ and 5′-GGTAGATGAGCTTCTGGGGA-3′, and RNA analysis was performed by TA cloning and sequencing, using pClone007 Versatile Simple Vector Kit (Beijing Tsingke Biotech Co., Ltd., Beijing, China).

### Conservative analysis and variant evaluation

National Center for Biotechnology Information Basic Local Alignment Search Tool (NCBI BLAST) was applied to evaluate the protein’s conservation, and the tertiary structures of wild-type and variant protein were conducted with the online SWISS-MODEL tool (https://swissmodel.expasy.org/) and further visualized using Visual Molecular Dynamics software (version 1.9.4a53) [28]. The variants were further classified following the American College of Medical Genetics and Genomics (ACMG) interpretation guidelines for sequence variants [29].

## Results

### Clinical findings

**Fig 2:**
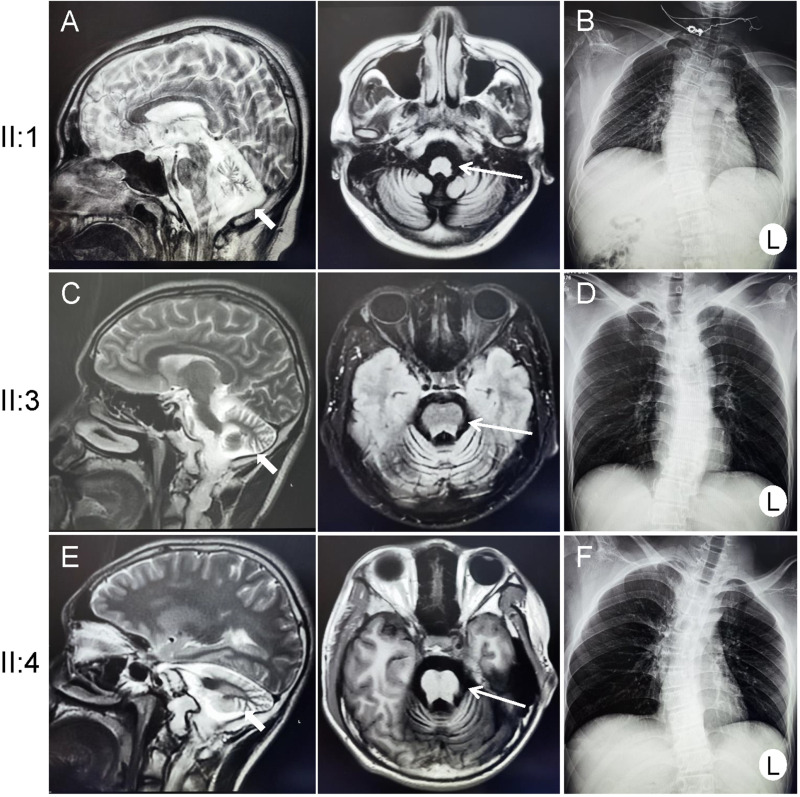
Radiological images of three patients (II:1, II:3, and II:4). Brain magnetic resonance imaging of three patients (**A**, **C**, and **E**) showing cerebellar atrophy (thick short arrow) and brain stem atrophy (thin long arrow), and chest X-ray showing scoliosis (**B**, **D**, and **F**).

Three patients (Fig 1A), including one female (II:1) and two males (II:3 and II:4), manifested similar abnormalities, having cerebellar ataxia, weak tendon reflexes, atrophy of distal muscles, slow neurological deterioration, craniofacial dysmorphism, enamel hypoplasia, osteopenia, chondrodysplasia punctata, scoliosis, pes cavus, and liver dysfunction (Table 1). Brain magnetic resonance imaging showed marked cerebellar atrophy and brain stem atrophy, and chest X-ray indicated scoliosis (Fig 2). Patient II:1 had walk instability and balance problems at about the age of 3 years with a slow progression, and was unable to walk independently at age 13. Patient II:3 had walk instability beginning at the age of 7 years, and his little brother (patient II:4) was found to have the walking difficulty at 2 years.

**Table 1:** Clinical features and examinations of four family members.

Subject	Mother I:2	Patient II:1	Patient II:3	Patient II:4
**Gender**	Female	Female	Male	Male
**Age at examination (years)**	55	32	29	15
**Age at onset (years)**	–	3	7	2
**Cerebellar ataxia**	No	Yes	Yes	Yes
**Weak tendon reflexes**	No	Yes	Yes	Yes
**Atrophy of distal muscles**	No	Yes	Yes	Yes
**Slow neurological deterioration**	No	Yes	Yes	Yes
**Craniofacial dysmorphism**	No	Yes	Yes	Yes
**Enamel hypoplasia**	No	Yes	Yes	Yes
**Osteopenia**	No	Yes	Yes	Yes
**Chondrodysplasia punctata**	No	Yes	Yes	Yes
**Scoliosis**	No	Yes	Yes	Yes
**Pes cavus**	No	Yes	Yes	Yes
**Liver dysfunction**	No	Yes (elevated total bile acid)	Yes (elevated total bilirubin and direct bilirubin)	No
**Brain anomaly (magnetic resonance imaging)**	No	Cerebellar and brain stem atrophy	Cerebellar and brain stem atrophy	Cerebellar and brain stem atrophy

### Variant analysis

The detailed exome sequencing data are shown in S1 Table. The novel compound heterozygous variants in the *PEX10* gene, including a novel splicing variant c.113-2A>G and a transition c.890T>C, shared in the two tested patients (II:1 and II:3), were considered as the potential pathogenic causes following the filtering processes and inheritance pattern. No further potential disease-associated variants, including homozygous or compound heterozygous variants, were found. Except the very low frequency (4.72×10^-5^) of c.890T>C variant in ChinaMAP, which is also deposited in the dbSNP (rs724160000), HGMD (CM090797), and ClinVar (ID 162432, interpreted as likely pathogenic or uncertain significance), the variants were absent in NHLBI ESP6500 and gnomAD, as well as Chinese controls (900 from our in-house exome database and 2375 from the BGI in-house database). The compound heterozygous variants were further validated in three patients by Sanger sequencing, and the heterozygous transition c.890T>C was also found in the unaffected mother (I:2) (Fig 1A–1C, Table 2). The compound heterozygous variants (c.113-2A>G and c.890T>C) co-segregated with the disease phenotype in the family. The novel c.113-2A>G variant was predicted to affect the splicing, by leading to the loss of canonical splice acceptor site. The recurrent c.890T>C variant was predicted to be damaging by *in silico* analyses (Table 2).

**Table 2:** Analysis of the *PEX10* variants identified in the PBDs family.

Items	Variant 1	Variant 2
**Reference sequence**	NG_008342.1, NM_153818.2, NP_722540.1	NM_153818.2, NP_722540.1
**Exon**	–	5
**Intron**	1	–
**Nucleotide change**	c.113-2A>G	c.890T>C
**Amino acid change**	p.Ala39_Gly65del	p.Leu297Pro
**Variant type**	Splicing variant	Missense variant
**Family members**	Patient II:1, II:3, and II:4	Mother (I:2) and patient II:1, II:3, and II:4
**dbSNP141**	No	rs724160000
**1000 Genomes Project**	No	No
**NHLBI ESP6500**	No	No
**gnomAD**	No	No
**ChinaMAP**	No	4.72×10^-5^
**In-house exome database**	No	No
**HGMD**	No	CM090797
**ClinVar**	No	Conflicting interpretations (ID 162432)
**PolyPhen-2**	–	Probably damaging
**FATHMM**	–	Damaging
**MutationAssessor**	–	High (functional)
**MutationTaster2021**	Deleterious (splice site lost)	Deleterious
**CADD (phred score)**	Deleterious (32)	Deleterious (28.8)
**BDGP NNSplice**	Destroy the acceptor site	–
**NetGene2**	Destroy the acceptor site	–
**SpliceAI (delta score)**	Acceptor loss (0.95)	–
**Pangolin (delta score)**	Splice loss (0.83)	–
**ACMG criteria applied**	Pathogenic (PVS1+PM2+PM3+PP1+PP3)	Pathogenic (PS3+PM2+PM3+PP1+PP3)

*PEX10*, the peroxisomal biogenesis factor 10 gene; PBDs, peroxisome biogenesis disorders; dbSNP, Single Nucleotide Polymorphism database; NHLBI ESP6500, National Heart, Lung, and Blood Institute Exome Sequencing Project 6500; gnomAD, Genome Aggregation Database; ChinaMAP, China Metabolic Analytics Project; HGMD, Human Gene Mutation Database; PolyPhen-2, Polymorphism Phenotyping version 2; FATHMM, Functional Analysis through Hidden Markov Models; CADD, Combined Annotation Dependent Depletion; BDGP NNSplice, Berkeley Drosophila Genome Project Splice Site Prediction by Neural Network; ACMG, American College of Medical Genetics and Genomics; PVS, pathogenic very strong; PM, pathogenic moderate; PP, pathogenic supporting; PS, pathogenic strong.

**Fig 3:**
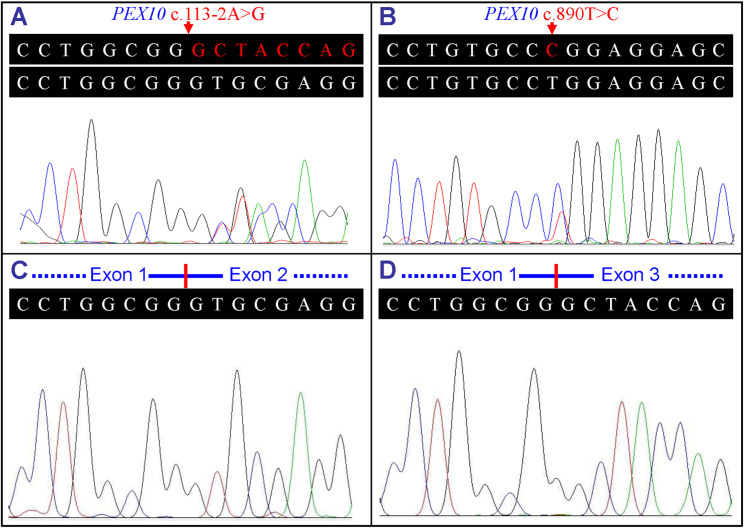
Sequence analysis of the cDNA in a family with PBDs. (**A**) cDNA sequence with *PEX10* c.113-2A>G variant of patient II:1. (**B**) cDNA sequence with *PEX10* c.890T>C variant of patient II:1. (**C**) cDNA sequence of the normal splicing in the *PEX10* gene of patient II:1. (**D**) cDNA sequence of the abnormal splicing caused by the *PEX10* c.113-2A>G variant of patient II:1. cDNA, complementary DNA; PBDs, peroxisome biogenesis disorders; *PEX10*, the peroxisomal biogenesis factor 10 gene.

By reverse transcription, PCR amplification, electrophoresis, and Sanger sequencing, the effects on RNA of identified compound heterozygous variants, c.113-2A>G (p.Ala39_Gly65del) and c.890T>C (p.Leu297Pro), were shown (Fig 3). The potential splicing variant c.113-2A>G was found to cause canonical splice acceptor site inactivation and exon 2 skipping, which can cause in-frame deletions (p.Ala39_Gly65del), a large portion of transmembrane region. No significant difference was shown in the electropherogram AUP for the wild-type (c.113-2A) and c.113-2A>G mutant alleles in three patients (*P* = 0.2140, S1 Fig). No correct splicing remained which was related to the variant c.113-2A>G (S2 Fig).

**Fig 4:**
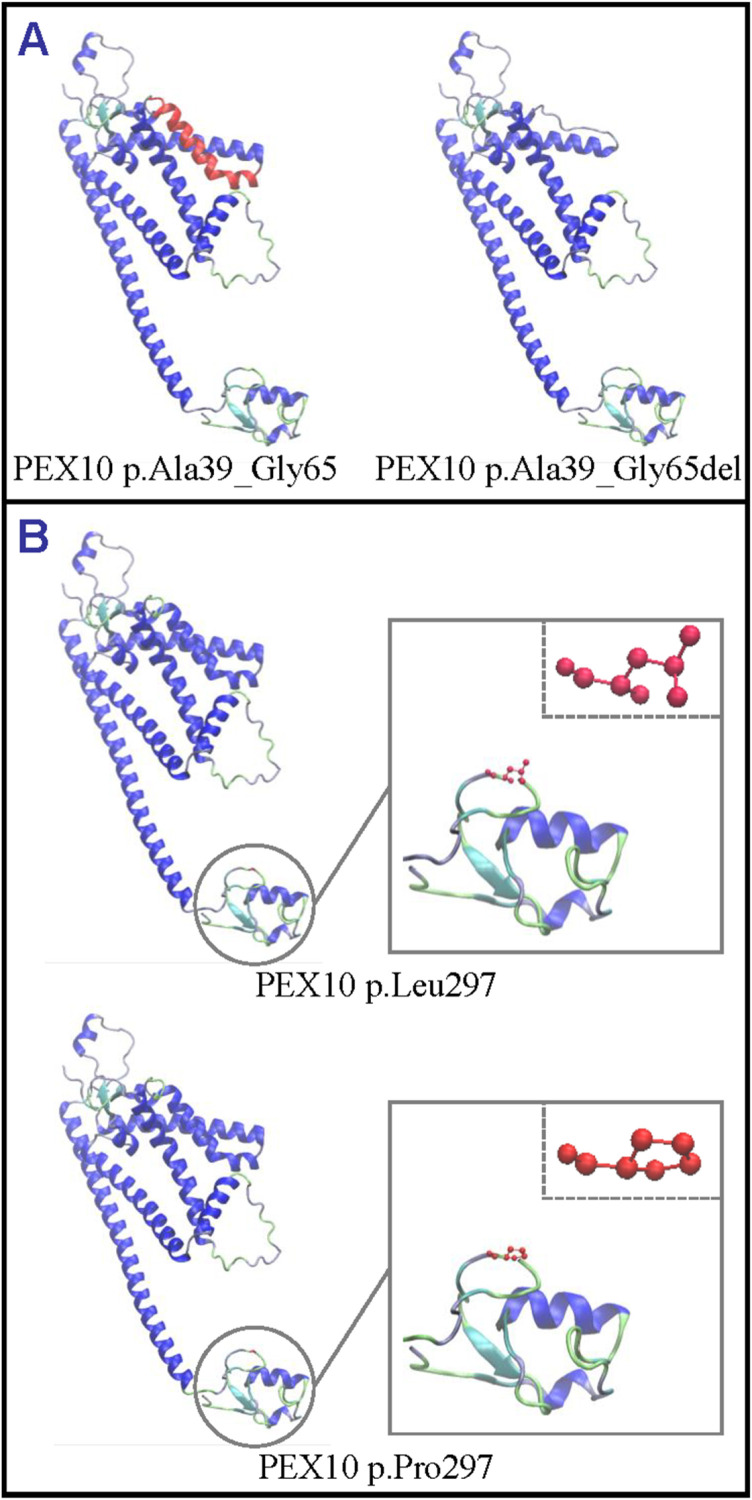
Cartoon model of PEX10 protein structure visualized by Visual Molecular Dynamics based on SWISS-MODEL modelling. (A) The deleted residues at position 39 to 65 (p.Ala39_Gly65del) are colored in the wild-type structure (p.Ala39_Gly65). (B) The residues at position 297, leucine (Leu) and proline (Pro), are indicated with ball-and-stick models. PEX10, peroxisomal biogenesis factor 10.

Sequence alignment of multiple orthologous proteins revealed that leucine at position 297 (p.Leu297) of the PEX10 protein, a functionally critical part of zinc-binding motif, near the zinc binding site (p.Cys296), was highly conservative in various organisms (Fig 1D). Structural modelling showed the conformational changes caused by the two variants (Fig 4). According to the ACMG variant interpretation guidelines, c.113-2A>G (p.Ala39_Gly65del) and c.890T>C (p.Leu297Pro) were deemed as “pathogenic”.

## Discussion

The *PEX10* pathogenic variants were reported to be responsible for two definite disorders, PBD6A and PBD6B, in Online Mendelian Inheritance in Man (OMIM) database, variable in clinical severity. PBD6A, a form of Zellweger syndrome, is characterized by neurodevelopmental abnormalities (neuronal migration defects, leukodystrophy, cognitive and psychomotor delay, profound dystonia, and seizures), skeletal abnormalities (craniofacial abnormalities and achondroplasia), and multisystem impairments (eyesight, hearing, liver, and kidney impairments), and the survival of affected infants is usually less than one year [7,15,30–32]. The features of PBD6B are milder than those of PBD6A, corresponding to the phenotypes of NALD and IRD, with a longer disease duration in the sufferers, as well as a slow progression and a later diagnosis [7,11].

The severity of PBDs was reported to be related to disease-causing gene variants which can cause changes in peroxidase function, matrix protein import capacity, and peroxisome numbers, via complete or partial loss-of-function alleles [9,14]. Due to the clinical and genetic overlaps of the subtypes of PBDs, this group of diseases can be divided into independent subtypes of PBDs by disease-causing genes, benefiting to the precise molecular genetic diagnosis [33]. The phenotype spectrum of the *PEX10*-related PBDs ranges from lethal to mild, and the clinical diagnosis include Zellweger syndrome, NALD, and IRD based on the onset age, symptoms, and signs [34,35]. Variants in important parts of the gene account for severe phenotypes of a certain disorder, while in non-critical parts, variants can result in milder subtypes or increase the disease susceptibility. Thus, based on the underpinning of shared genetic etiology, milder diseases can be classified as less severe subtypes of severe phenotype-related disorders (e.g., *PEX10*-related PBDs) that exhibit different expressivity [33].

This study identified compound heterozygous variants c.113-2A>G (p.Ala39_Gly65del) and c.890T>C (p.Leu297Pro) in the *PEX10* gene in a non-consanguineous Han-Chinese family with PBDs, which co-segregated with the disease phenotype. The deceased father (I:1) was supposed to be an obligate heterozygote of c.113-2A>G variant. In addition to the absence of c.113-2A>G variant in public and in-house exome databases, and a low frequency of c.890T>C variant in public databases, the *in silico* predicted deleterious effect and RNA analysis further supported that both variants were pathogenic factors for PBDs in our family. The splicing variant c.113-2A>G may not induce nonsense-mediated mRNA decay, as well as retaining correct splicing. Our three patients have the milder presentations of PBDs, and the clinical features include cerebellar ataxia, weak tendon reflexes, atrophy of distal muscles, slow neurological deterioration, craniofacial dysmorphism, enamel hypoplasia, osteopenia, chondrodysplasia punctata, scoliosis, and pes cavus, which are consistent with the reported manifestations of PBD6B cases [7,35–37].

The *PEX10* gene, located on chromosome 1q36.32, consisting of 6 exons, can encode an integral peroxisomal membrane protein, which is known as a multi-pass membrane protein with a zinc-binding motif in the C-terminus [11,36]. There are two main transcripts due to the alterative splice acceptor site in intron 3, leading to the 346-amino acid (39.2-kDa) and 326-amino acid (37-kDa) isoform, in which the shorter one may be more abundant and the identified substitution was previously reported as c.830T>C (p.Leu277Pro) [11,35,38]. In drosophila, *pex10* variants had peroxisomal protein import defect, increased very-long-chain fatty acids levels, and growth restriction, similar to features of PBDs in humans [39]. *Pex10*^*Cys294Tyr/Cys294Tyr*^ mice displayed neurological deficits with progressive locomotion defects, peroxisomal biochemical abnormalities, and neonatal death [40].

To date, at least 34 *PEX10* gene variants, in the homozygous or compound heterozygous state, have been reported to be responsible for PBDs in published literature and in the HGMD, as well as our study. A total of 13 missense, 9 frameshift (including 4 small insertions, 4 small deletions, and 1 small indel), 7 nonsense, and 2 splicing variants, as well as 3 variants leading to the loss of the start codon, were identified in 60 cases. Variants are mainly in exon 3 (11/34) and involving in 5 coding exons (Table 3) [7,41–47]. The homozygous c.874_875del (p.Leu292Valfs*66) variant is the most common reported variant (15/30 homozygotes, 32/120 alleles), and the c.764dup (p.Leu256Alafs*103) variant is the second one (10/120 alleles). Genotype-phenotype association may be revealed for the *PEX10* deficiency, which can be partially veiled by the limited PBD phenotype description in the literature. Generally, in homozygotes, missense variant, p.(Leu177Arg), retaining residual function, is related to milder phenotypes (i.e., PBD6B), and missense variant, p.(Cys296Phe), interrupting the formation of critical C-terminal zinc-binding domain, and truncations relating to splicing, nonsense, and frameshift variants, c.600+1G>A (p.Gly65Alafs*36), p.Arg264*, and p.Leu292Valfs*66, affecting large portions of the coding region, are associated with severe phenotypes (i.e., PBD6A) [5–7,9,38,43,48]. Compound heterozygotes, modifier genes, and other confounding factors can complicate the genotype-phenotype relationship [5,7,11,14,32,35–37,43–48]. In our study, the novel identified splicing variant c.113-2A>G led to exon 2 skipping and the loss of transmembrane region (p.Ala39_Gly65del). The identified missense variant c.890T>C (p.Leu297Pro), with hydrophobic property, involving a functionally critical part of zinc-binding motif, near the zinc binding site (p.Cys296), was previously reported in two unrelated patients, responsible for the milder phenotype of PBDs (i.e., PBD6B) in a compound heterozygous state with a missense variant, c.209G>A, p.(Gly70Glu), and a frameshift variant, c.337del, p.(Leu113Trpfs*40), respectively [7,35]. The compound heterozygous variants may lead to the milder phenotype, PBD6B, in our family via a loss-of-function mechanism (predicted by LoGoFunc predictor in https://itanlab.shinyapps.io/goflof/) [49]. Due to the autosomal recessive inheritance pattern, newborn screening for PBDs or prenatal genetic testing for at-risk fetus, or even preimplantation genetic testing, is recommended, especially for those with affected siblings [50,51]. It is a pity that peroxisomal functional studies were not performed in this study. Further studies like peroxisomal activity in patient-related materials and variants’ functional study may reveal the exact pathogenicity and disease mechanism [14,17].

**Table 3:** The reported variants in the *PEX10* gene associated with PBDs.

No.	Number of cases	Nucleotide change^a^	Amino acid change^a^	Location	Variant status	Provenance	Variant type	Diagnosis^b^	References
**1**	1	c.1A>G, c.199C>T	p.(Met1?), p.(Gln67*)	Exon 1, exon 3	Compound heterozygote	NA	Start-loss, nonsense	PBD6B	[32]
**2**	1	c.1A>G, c.463C>T	p.(Met1?), p.(Gln155*)	Exon 1, exon 3	Compound heterozygote	Albania	Start-loss, nonsense	PBD6B	[44]
**3**	1	c.2T>C, c.790C>T	p.(Met1?), p.(Arg264*)	Exon 1, exon 4	Compound heterozygote	NA	Start-loss, nonsense	PBD6B	[37]
**4**	3	c.2T>C, c.980G>A	p.(Met1?), p.(Cys327Tyr)	Exon 1, exon 6	Compound heterozygote	NA	Start-loss, missense	PBD6B	[46]
**5**	1^c^	c.2T>C	p.(Met1?)	Exon 1	Heterozygote	NA	Start-loss	PBD	[41]
**6**	1^c^	c.3G>A	p.(Met1?)	Exon 1	Heterozygote	NA	Start-loss	PBD	[41]
**7**	1	c.4del, c.895G>T	p.(Ala2Profs*10), p.(Glu299*)	Exon 1, exon 5	Compound heterozygote	NA	Frameshift, nonsense	PBD	[42]
**8**	1	c.13_28delinsCCGCCAGCACCTGCGCCGCC, c.764dup	p.Ala5Profs*47, p.Leu256Alafs*103	Exon 1, exon 4	Compound heterozygote	NA	Frameshift	PBD	[17]
**9**	1	c.26dup, c.874_875del	p.(Glu10Glyfs*41), p.(Leu292Valfs*66)	Exon 1, exon 5	Compound heterozygote	NA	Frameshift	PBD6B	[45]
**10**	2	c.28dup	p.(Glu10Glyfs*41)	Exon 1	Homozygote	NA	Frameshift	PBD6B	[47]
**11**	3	c.113-2A>G, c.890T>C	p.Ala39_Gly65del, p.Leu297Pro	Intron 1, exon 5	Compound heterozygote	China	Splicing, missense	PBD6B	This study
**12**	1^c^	c.203C>A	p.(Thr68Asn)	Exon 3	Heterozygote	NA	Missense	PBD	[41]
**13**	1^c^	c.208G>C	p.(Gly70Arg)	Exon 3	Heterozygote	NA	Missense	PBD	[41]
**14**	1	c.209G>A, c.890T>C	p.(Gly70Glu), p.(Leu297Pro)	Exon 3, exon 5	Compound heterozygote	China	Missense	PBD6B	[7]
**15**	1	c.211G>A	p.(Glu71Lys)	Exon 3	Homozygote	NA	Missense	PBD	[41]
**16**	1	c.233A>G	p.(Gln78Arg)	Exon 3	Homozygote	NA	Missense	PBD	[41]
**17**	1	c.337del, c.890T>C	p.(Leu113Trpfs*40), p.(Leu297Pro)	Exon 3, exon 5	Compound heterozygote	NA	Frameshift, missense	PBD6B	[35]
**18**	1	c.352C>T	p.(Gln118*)	Exon 3	Homozygote	NA	Nonsense	PBD	[41]
**19**	1	c.373C>T, c.930C>G	p.Arg125*, p.His310Gln	Exon 3, exon 5	Compound heterozygote	NA	Nonsense, missense	PBD6B	[5]
**20**	1	c.530T>G	p.(Leu177Arg)	Exon 3	Homozygote	Sweden	Missense	PBD6B	[48]
**21**	1	c.600+1G>A	p.Gly65Alafs*36	Intron 3	Homozygote	NA	Splicing	PBD6A	[5]
**22**	1	c.600+1G>A	p.(Gly65Alafs*36)	Intron 3	Homozygote	NA	Splicing	PBD	[41]
**23**	1	c.697del	p.(Ala233Profs*131)	Exon 4	Homozygote	NA	Frameshift	PBD	[41]
**24**	1	c.706dup	p.(Ser236Lysfs*123)	Exon 4	Homozygote	NA	Frameshift	PBD	[41]
**25**	2	c.764dup	p.(Leu256Alafs*103)	Exon 4	Homozygote	NA	Frameshift	PBD	[41]
**26**	2^c^	c.764dup	p.(Leu256Alafs*103)	Exon 4	Heterozygote	NA	Frameshift	PBD	[41]
**27**	1	c.764dup, c.790C>T	p.(Leu256Alafs*103), p.(Arg264*)	Exon 4	Compound heterozygote	NA	Frameshift, nonsense	PBD	[42]
**28**	1	c.764dup, c.928C>G	p.(Leu256Alafs*103), p.(His310Asp)	Exon 4, exon 5	Compound heterozygote	NA	Frameshift, missense	PBD	[42]
**29**	1	c.764dup, c.992G>A	p.(Leu256Alafs*103), p.(Arg331Gln)	Exon 4, exon 6	Compound heterozygote	NA	Frameshift, missense	PBD6B	[37]
**30**	1	c.790C>T	p.Arg264*	Exon 4	Homozygote	Turkey	Nonsense	PBD6A	[9]
**31**	1	c.790C>T, c.941G>A	p.Arg264*, p.Trp314*	Exon 4, exon 5	Compound heterozygote	Germany	Nonsense	PBD	[9]
**32**	1^c^	c.790C>T	p.(Arg264*)	Exon 4	Heterozygote	NA	Nonsense	PBD	[41]
**33**	1	c.874_875del	p.Leu292Valfs*66	Exon 5	Homozygote	Japan	Frameshift	PBD6A	[38]
**34**	2	c.874_875del	p.Leu292Valfs*66	Exon 5	Homozygote	NA	Frameshift	PBD	[17]
**35**	11	c.874_875del	p.Leu292Valfs*66	Exon 5	Homozygote	Japan	Frameshift	PBD6A	[6]
**36**	1	c.874_875del	p.(Leu292Valfs*66)	Exon 5	Homozygote	NA	Frameshift	PBD	[41]
**37**	1^c^	c.874_875del	p.(Leu292Valfs*66)	Exon 5	Heterozygote	NA	Frameshift	PBD	[41]
**38**	1	c.887G>T	p.(Cys296Phe)	Exon 5	Homozygote	Turkey	Missense	PBD6A	[43]
**39**	3	c.887G>T, c.992G>A	p.(Cys296Phe), p.(Arg331Gln)	Exon 5, exon 6	Compound heterozygote	France	Missense	PBD6B	[36]
**40**	1^c^	c.895G>T	p.(Glu299*)	Exon 5	Heterozygote	NA	Nonsense	PBD	[41]
**41**	1	c.979T>C	p.(Cys327Arg)	Exon 6	Homozygote	NA	Missense	PBD	[41]
**42**	1^c^	c.992G>A	p.(Arg331Gln)	Exon 6	Heterozygote	NA	Missense	PBD	[41]

*PEX10*, the peroxisomal biogenesis factor 10 gene; PBD, peroxisome biogenesis disorder; NA, not available.

^a^The description of variants not conforming to current naming conventions is recalibrated following the Human Genome Variation Society nomenclature (https://varnomen.hgvs.org/) using the reference sequence (NG_008342.1, NM_153818.2, and NP_722540.1).

^b^The diagnosis determined as PBD means that the clinical phenotypes are not detailed in the reported reference.

^c^Eight cases (7 compound heterozygotes and 1 heterozygote) with 10 heterozygous variants were not detailed in the reference [41].

## Conclusions

In summary, compound heterozygous *PEX10* variants, c.113-2A>G (p.Ala39_Gly65del) and c.890T>C (p.Leu297Pro), were likely the responsible variants for the multisystem abnormalities in a Han-Chinese family with PBD6B, the milder subtype of the *PEX10*-related PBDs. Exome sequencing provides a cost-effective and expedited approach to identify pathogenic variants responsible for highly heterogeneous disorders. This is the first known report of *PEX10* c.113-2A>G variant, with the exon 2-skipping effect first confirmed by RNA analysis. Our finding may provide new insights and approaches into the genetic cause and diagnosis of PBDs, and may also have implications for genetic counseling and clinical intervention. Because of the limited number of patients in this study and the relative rarity of the disease, more cases confirmed by genetic analysis in other families and the evaluation of gene variants’ pathogenicity are warranted. More in-depth research including variants’ functional analyses *in vitro* and animal models with genetic defects *in vivo*, and the discovery of variants in the affected families will help to discover the cause and provide assistance for gene therapy of PBDs intervention.

## Supporting information

S1 Table The summarized exome sequencing data of the two patients. (PDF)

S1 Fig RNA analysis of *PEX10* c.113-2A>G variant. (PDF)

S2 Fig RNA analysis of *PEX10* c.113-2A>G variant and c.890T>C variant. (PDF)
