# Supplementary material for: Identification of novel compound heterozygous variants in the PEX10 gene in a Han-Chinese family with PEX10-related peroxisome biogenesis disorders
Source: PLoS One. 2025 Apr 23;20(4):e0322137. doi: 10.1371/journal.pone.0322137 (PMC12017559; doi:10.1371/journal.pone.0322137)
Supplement: S1 Table — (PDF) [file pone.0322137.s001.pdf]

S1 Table. The summarized exome sequencing data of the two patients.

Data	Patient II:1	Patient II:3
**Raw bases (Mb)**	17,763.10	15,239.16
**Clean bases (Mb)**	17,432.97	15,125.16
**Mapping rate on genome**	99.98%	99.99%
**Average sequencing depth on target**	148.69	131.45
**Target covered ≥20×**	98.44%	98.25%
**Total SNPs**	130,247	131,725
**Total indels**	21,145	21,903

Mb, megabase; SNPs, single nucleotide polymorphisms; indels, insertions-deletions.
