# Supplementary material for: Identification of novel compound heterozygous variants in the PEX10 gene in a Han-Chinese family with PEX10-related peroxisome biogenesis disorders
Source: PLoS One. 2025 Apr 23;20(4):e0322137. doi: 10.1371/journal.pone.0322137 (PMC12017559; doi:10.1371/journal.pone.0322137)

S1 Fig. RNA analysis of *PEX10* c.113-2A>G variant. The areas under the peaks of wild-type c.113-2A and c.113-2A>G mutant alleles by sequencing analysis of *PEX10* complementary DNA from three patients (II:1, II:3, and II:4). *PEX10*, the peroxisomal biogenesis factor 10 gene; ns, not significant.

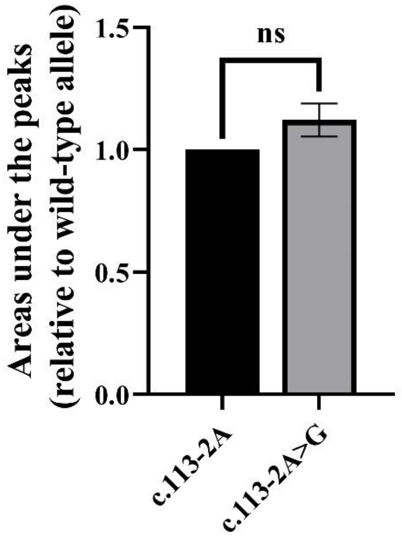

Supplement: S1 Fig — (PDF) [file pone.0322137.s002.pdf]
