# Supplementary material for: Identification of novel compound heterozygous variants in the PEX10 gene in a Han-Chinese family with PEX10-related peroxisome biogenesis disorders
Source: PLoS One. 2025 Apr 23;20(4):e0322137. doi: 10.1371/journal.pone.0322137 (PMC12017559; doi:10.1371/journal.pone.0322137)

S2 Fig. RNA analysis of *PEX10* c.113-2A>G variant and c.890T>C variant. (**A**) cDNA sequence of the abnormal splicing caused by the *PEX10* c.113-2A>G variant and wild-type c.890T of patient II:1. (**B**) cDNA sequence of the normal splicing and mutant c.890C in the *PEX10* gene of patient II:1. *PEX10*, the peroxisomal biogenesis factor 10 gene; cDNA, complementary DNA.

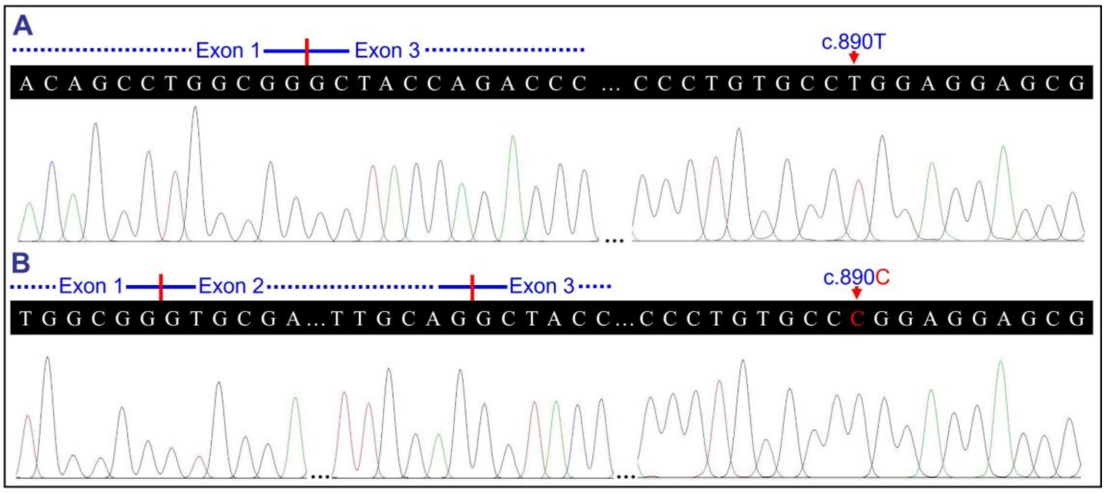

Supplement: S2 Fig — (PDF) [file pone.0322137.s003.pdf]
